# Environmental Sustainability and Mold Hygiene in Buildings

**DOI:** 10.3390/ijerph15040681

**Published:** 2018-04-04

**Authors:** Haoxiang Wu, Tsz Wai Ng, Jonathan WC Wong, Ka Man Lai

**Affiliations:** 1 Department of Biology, Hong Kong Baptist University, Kowloon Tong, Hong Kong, China; kubeng@life.hkbu.edu.hk (H.W.); wai64@yahoo.com.hk (T.W.N.); jwcwong@hkbu.edu.hk (J.W.C.W.); 2 Hong Kong Baptist University Sino-Forest Applied Research Centre for Pearl River Delta Environment (ARCPE), Kowloon Tong, Hong Kong, China

**Keywords:** mold, hygiene, sustainability, built environments, air-conditioning

## Abstract

Environmental sustainability is one of the key issues in building management. In Hong Kong, one of the initiatives is to reduce the operation hours of air-conditioning in buildings to cut down energy consumption. In this study, we reported a mold contamination case in a newly refurbished laboratory, in which the air-conditioner was switched from 24- to 18-h mode after refurbishment. In order to prevent mold recurrence, the air-conditioner was switched back to 24-h mode in the laboratory. During the mold investigation, visible mold patches in the laboratory were searched and then cultured, counted and identified. Building and environmental conditions were recorded, and used to deduce different causes of mold contamination. Eight contaminated sites including a wall, a bench, some metal and plastic surfaces and seven types of molds including two *Cladosporium* spp., two *Aspergillus* spp., one *Rhizopus* sp., one *Trichoderma* sp., and one *Tritirachium* sp. were identified. *Cladosporium* spp. were the most abundant and frequently found molds in the laboratory. The contaminated areas could have one to five different species on them. Based on the mold and environmental conditions, several scenarios causing the mold contamination were deduced, and different mold control measures were discussed to compare them with the current solution of using 24-h air-conditioning to control mold growth. This study highlights the importance of mold hygiene in sustainable building management.

## 1. Introduction

Environmental sustainability is one of the key issues in building management. According to the World Green Building Council (WGBC), one of the features of green buildings is efficient use of energy, water and other resources, which could not only save operation costs of the building, but also give consideration to environmental sustainability [1]. In Hong Kong, electricity consumption for air-conditioners accounts for 50% of electricity consumption in commercial buildings and 30% of total electricity consumption [2]. Hence, saving energy by cutting down the operation hours or turning up the set temperature of air-conditioners can obviously help towards building sustainability; a drop of 1 °C in the room temperature consumes 3% more energy [3].

As part of a university overseeing a large building portfolio, the building and facilities management team is responsible for setting up policy to improve building sustainability. In light of the concern for energy saving, new initiatives related to the use of air-conditioners are regularly proposed and tested to further enhance the environmental performance of buildings in the campus. For instance, one of the initiatives is to reduce the operation of the air-conditioning system in some general laboratories from 24 to 18 h or even less time. However, after running this initiative, several mold contamination cases were reported. In general, mold requires the basic elements of water, nutrition and proper temperature for growth [4,5]. It is likely that this energy saving initiative has changed some of these elements and thereby leads to mold contamination. The health effects related to indoor mold contamination have been reviewed by the World Health Organization (WHO) and other organizations [6,7,8]. In brief, some indoor molds can be pathogenic, and are able to produce allergens, mycotoxins and other hazards [6]. The mold patches and the moldy smells (due to the emission of various volatile organic compounds) may also cause discomfort to the occupants and reduce work productivity as well as increase building management costs on maintenance and remediation. Therefore, it is important to prevent mold growth, clean up the mold contamination as soon as it happens, and stop its recurrence as a good hygiene practice. 

Currently, in the university campus, one of the measures to prevent mold contamination in laboratories and other environments, which is not due to water leakage, is to turn on the air-conditioning unit continuously to reduce the room relative humidity (RH) and temperature to slow down the mold activity. This mold control measure is common because no other work or instrument is needed to prevent mold growth. However, this may not be the most sustainable way to balance the energy consumption and mold hygiene. 

Therefore, the aim of this study is to use this mold contamination case as an example to highlight the importance of mold hygiene assessment in building design and prior to any environmental alteration. Moreover, if mold contamination does occur, investigating the cause of contamination could help predict the mold speciation and signal potential health risks. Strategically focusing on fixing the physical environment could be a more sustainable alternative than just relying on air-conditioning to control mold growth. High humidity and hot climate in tropical and subtropical regions are favorable for mold growth. The outcome of this study may facilitate future mold remediation work and provide alternative measures, which are sustainable to maintain a good mold hygiene environment. 

## 2. Materials and Methods

### 2.1. Sampling and Cultivation

**Figure 1:**
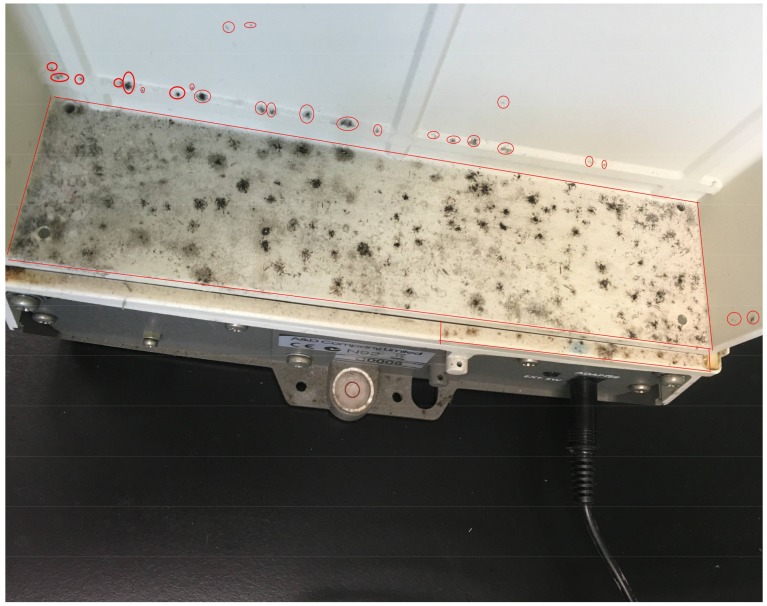
Mold patches behind an analytical balance (M008). The red circles indicate individual mold colonies. The red rectangular area represents one mold patch area because individual colonies are numerous and cannot be separated.

Several visual inspections for mold contamination were conducted by two researchers to locate the contaminated areas in a laboratory in September 2016. Environmental mold samples were collected from each contaminated area by swabbing the entire representative mold patches. The location of the contaminated site/object in the laboratory, the total mold patch area, the type of surface material, whether the site/object has been previously cleaned and if visible dust was present on the site/object were recorded. Figure 1 shows mold contamination at the back of an analytical balance (M008) and the mold patch areas as an example. Where mold patches were difficult to separate from each other, the entire contaminated area was measured as one patch, the rectangular space shown in Figure 1 as compared to the individual red circles (individual colonies). In some areas, the mold colonies were not obvious and could not be clearly identified from the dust. In this case, the dust area was determined and sampled in the same way as the mold patch. 

The interior layout, position of the air supply and return air of the air-conditioning units and temperature of the two adjacent rooms as well as temperature outside the laboratory door were recorded. The surface temperature and water activity (a_w_) of the contaminated areas were measured using a Hygropalm 23 & probe type HC2-AW (Rotronic AG, Crawley, UK). The room RH and temperature at the time of mold sampling were taken simultaneously using a hygrometer. The light of the mold-contaminated surface was rated according to four levels: (1) room light + direct sunlight, (2) room light, (3) dim and (4) dark. The position of the site/object was also classified as in open or hidden space. Lastly, the pressure difference between the laboratory and corridor was also measured using a pocket manometer (Furness Controls Ltd., Bexhill-on-Sea, UK).

After sampling and field investigation, the mold species on the swab samples were extracted with sterilized deionized (DI) water using a vortex, and then serially diluted before plate counting on potato dextrose agar (PDA) after incubation at 28 °C for 4 days to estimate the abundance and diversity of the molds. 

### 2.2. Species Identification

In order to facilitate the mold identification, colonies with different morphology were separated and sub-cultured on PDA plates until sporulation. The spore forming structure was examined under a microscope to identify the mold to the genus level, if possible, to narrow down the selection of species-specific primers for further identification [9,10]. Genomic DNA was extracted from the mycelia using a lysing matrix E tube (MP Biomedicals, Eschwege, Germany) and QIAamp DNA Mini Kit (Qiagen, Hilden, Germany) following the protocols suggested by the manufacturer. 

The extracted DNA samples that did not match with the selected primers were then multiplied using primers ITS4 (5′-TCC TCC GCT TAT TGA TAT GC-3′) and ITS5 (5′-GGA AGT AAA AGT CGT AAC AAG G-3′), and subsequently sequenced on a Life Tech 3730xl DNA Analyzer (Thermo Fisher, Waltham, MA, USA) by the Beijing Genomics Institute, BGI Co., Ltd. (Shenzhen, China). The DNA sequence was then aligned with NCBI’s GenBank sequence database using a BLAST search with 99.99% of similarity, and the species identity was further validated using species-specific primers, if available in the US Environmental Protection Agency (USEPA) list of common indoor molds [9,10]. Primers used in this study are listed in Table 1.

### 2.3. Mold Contamination Scenarios

The mold speciation is an indication of the diversity of the physical environment (e.g., water and nutrient levels) that has occurred or is still present in the contaminated area because different molds have different ecological niches and habitats. Combining the mold information and the on-site environmental features, different contamination scenarios were deduced.

Dew point was calculated to predict the temperature and RH conditions that cause condensation [11]. Other experimental data and models for mold growth, e.g., the mold growth prediction model developed by the Technical Research Center of Finland (VTT) and Tampere University of Technology in Ojanen et al.’s study [5] were referenced to help deduce the mold contamination scenarios [5].

## 3. Results

### 3.1. Mold Contaminated Areas and Environmental Conditions

**Figure 2:**
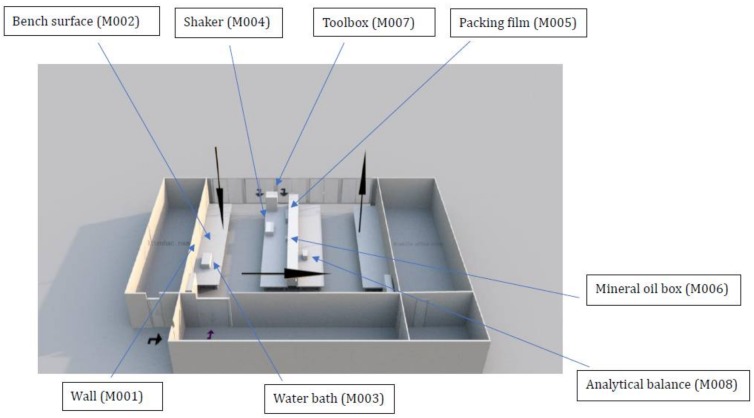
Laboratory layout and sample sites. The arrows in the laboratory indicate the air flow direction. The downward arrow on the left indicates the supply air coming out from the air diffusers and the upward arrow on the right points to the direction of the return air into the air-cooling units. The arrows shown outside the laboratory indicate the movement of the air into the laboratory. The room on the left of the laboratory is for the lab attendant and on right for the scientific officers. There are windows at the back of the laboratory. The windows are always kept closed.

**Figure 3:**
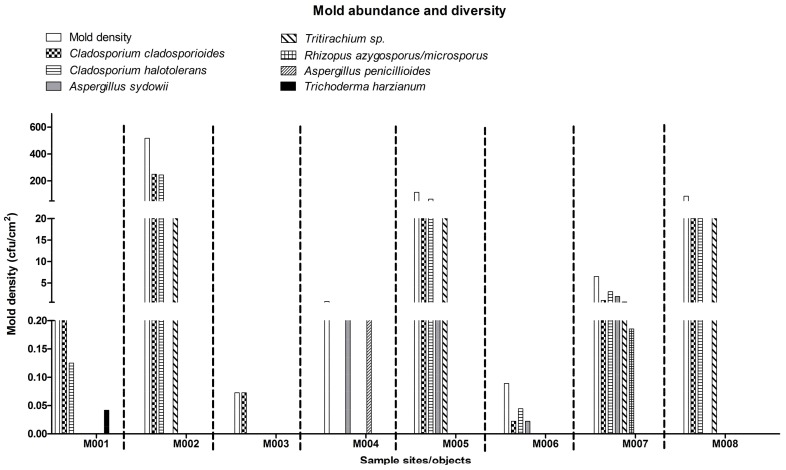
Mold abundance and diversity in each sample site/object.

Information about the contaminated areas and their environmental conditions are presented in Figure 2 and Table 2. In total, eight contaminated areas were identified, and all the contaminated surfaces had a higher a_w_ than the room RH (60.5%) in the laboratory. The contaminated areas were mainly located on the left side of the laboratory where the cooled supply air (according to the facilities management data, temperature and RH of the supply air were generally around 16 °C and 60%, respectively under similar environmental conditions) circulated before returning to the air-conditioner on the right side of the laboratory (Figure 2). The culturable mold abundance (in terms of mold density, colony-forming unit (CFU)/cm^2^) and diversity in each contaminated area is shown in Figure 3. In aggregate, seven species were isolated—two *Cladosporium* spp. (*Cladosporium cladosporioides* and *Cladosporium halotolerans*), two *Aspergillus* spp. (*Aspergillus penicillioides* and *Aspergillus sydowii*), *Rhizopus azygosporus/microsporus*, *Tritirachium* sp., and *Trichoderma harzianum*. Except for *C. halotolerans* and *Tritirachium*, all other molds were matched with their corresponding species-specific primers (Table 1).

A total of 120 cm^2^ mold patch area was observed on the anti-mold painted cement brick wall (M001). The wall surface had the highest a_w_ of 0.75 among other contaminated areas. The surface temperature was 21.8 °C as compared to 22.0 °C in the room (Table 2). The light level on the wall surface was dim because the wall is under a wall cabinet (Figure 2). According to the laboratory user, a larger contaminated surface than that we reported was observed before cleaning with diluted household chlorine bleach about 2–3 months ago.

According to the Estate Office, an anti-mold paint was applied onto the wall, but our results show that this paint could not guarantee a mold free environment. The mold density was relatively low (0.38 CFU/cm^2^) as compared to other areas (Figure 3). *C. cladosporioides* (56%), followed by *C. halotolerans* (33%) and *T. harzianum* (11%) were isolated in this area. The room behind the wall is occupied by a laboratory attendant. The attendant’s room is also used for storage of laboratory consumables and equipment. Due to its functional purpose, this room is always air-conditioned to maintain a stable temperature at 22.0 °C. The door of the laboratory is also located on this side and faces a corridor that is used by the laboratory and office users. The door of the corridor opens to the outdoor environment. At the time of the investigation, the RH of the corridor outside the laboratory was 96.0% and temperature was 24.0 °C. The air pressure in the corridor was 5 Pa higher than that in the laboratory indicating air infiltrated from the corridor into the laboratory. 

Part of the bench surface (M002) close to the contaminated wall (M001) was infested as well (400 cm^2^) with the second highest a_w_ of 0.74 and surface temperature of 21.7 °C (Table 2). The bench surface was dusty and had the highest mold density (>500 CFU/cm^2^). It was dominated by *C. halotolerans* and *C. cladosporioides* (48% and 47%, respectively; 5% *Tritirachium* sp.) (Figure 3).

Two metal surfaces, water bath (M003) and shaker (M004) had the lowest surface temperature of 20.1 and 19.8 °C, respectively (Table 2). Both surfaces had a water activity level of 0.71. These instruments were located on the left and middle benches and received a similar room light level (Figure 2). The water bath was used occasionally and thus dusty. The molds on the metal surface had been cleaned with bleach before but molds were found again at the time of sampling. The mold density was the lowest (0.07 CFU/cm^2^), and the total mold patch area was the smallest, 69 cm^2^, about 1% of the total water bath surface area (Figure 3). *C. cladosporioides* was the only species isolated. Two species of molds, *A. sydowii* (50%) and *A. penicillioides* (50%) were found in the 150 cm^2^ of total mold patch area on the shaker (M004) (about 2% of the total shaker surface). The mold density was about 0.67 CFU/cm^2^, a mid-range level compared to other areas. 

The other two contaminated objects near the shaker were the packing film (plastic material) wrapped around a Benchguard box (M005) and a plastic mineral oil box (M006). The packing film had a water activity of 0.69 and surface temperature of 21.3 °C (Table 2). It was hidden under a cupboard and had never been used, hence it was very dusty. The packing film had the largest mold patch area (1100 cm^2^, >20% of the total film surface) and the second highest mold density (114 CFU/cm^2^). Isolates included *C. halotolerans* (55.5%), *C. cladosporioides* (20%), *Tritirachium* sp. (24%) and *A. sydowii* (0.5%) (Figure 3).

The mineral oil box (M006) had a similar a_w_ (0.69) and temperature (21.4 °C) as the packing film (Table 2). It was placed in the same cupboard as the packing film and was dusty. A low mold density (0.09 CFU/cm^2^) with 3 species (50% *C. cladosporioides*, 25% *C. halotolerans* and 25% *A. sydowii*) was recorded in the 225 cm^2^ of mold patch area, more than 27% of the total surface (Figure 3).

A plastic toolbox (M007) located the back of a bench and next to a window was also contaminated by molds on the top surface (Figure 2). Due to its placement, the toolbox received room light and sunlight. The box was covered by dust since it was rarely used. Its a_w_ was 0.73 and the temperature was 21.1 °C (Table 2). The mold density was about 6 CFU/cm^2^ in the 270 cm^2^ sampled area. The highest number of molds (5 species) was isolated including *C. halotolerans* (46%), *C. cladosporioides* (14%), *A. sydowii* (28.5%), *Tritirachium* sp. (8.5%) and *R. azygosporus/microsporus* (3%) (Figure 3).

Finally, molds contaminated the back of an analytical balance (M008). The balance was the only mold-contaminated item on the right side of the laboratory (Figure 2). It had the lowest a_w_ (0.65 a_w_), and received little light exposure (Table 2). The mold density was approximately 86 CFU/cm^2^ covering 70 cm^2^, around 3% of the total surface. *Tritirachium* sp. was the most dominant species, which accounted for more than 50% of the total mold count (43 CFU/cm^2^), and followed by *C. halotolerans* (26%) and *C. cladosporioides* (23%) (Figure 3). On the right side of the laboratory, there is an office for 10 people and the air conditioning was operated in the same pattern as the laboratory (18 h operation and turned off throughout the weekend). 

### 3.2. Mold Occurrence Percentage

**Figure 4:**
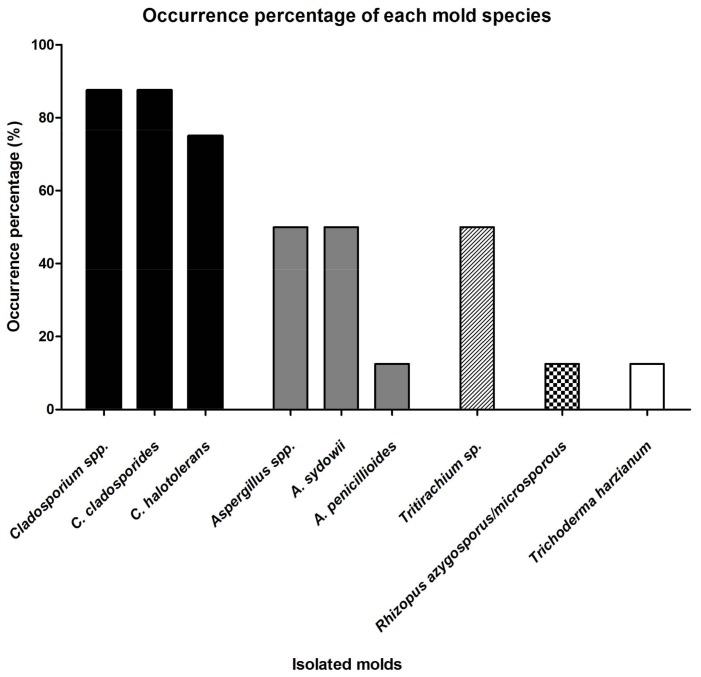
Occurrence percentage of each mold species.

Figure 4 summarizes the occurrence percentage of the isolated molds in the laboratory. Both *Cladosporium* spp. had higher occurrence percentage than other species in the laboratory, where *C. cladosporioides* was 88% (seven out of eight areas) and *C. halotolerans* was 75% (six out of eight areas). 

*A. sydowii* was present in four areas but its population on the packing film (M004) was very low, while *A. penicillioides* was found in the shaker (M004) only and contributed half of the population on this contaminated area with *A. sydowii*. *Tritirachium* sp. appeared on four areas, and its occurrence always coincided with *C. cladosporioides* and *C. halotolerans*. Its population level was the highest on the balance (M008) compared to other areas (50%). Together with *A. penicillioides*, *R. azygosporus/microsporus* and *T. harzianum* were present in only one contaminated area and their population levels were low.

### 3.3. Ecological Niches and Growth Requirements of the Isolated Molds

Table 3 presents the ecological niches and growth requirements of the isolated species which provided information to develop the mold contamination scenarios in each contaminated area. *Cladosporium* spp. are regarded as secondary colonizers in indoor environments based on their a_w_ requirement of 0.8–0.9 [12]. *C. cladosporioides* requires a high level of organic matter for growth as it is frequently found in dust, wallpaper and painted walls [13,14] and can tolerate cold environments [15]. *C. halotolerans* is reported to be halotolerant and able to grow under a low nutrient level as well as psychrotolerant [16]. It was reported that *C. halotolerans* adapts to the fluctuation of low a_w_ better than *Aspergillus niger* and *Penicillium rubens* in indoor environmental conditions [17].

*Aspergillus* spp. are recognized as primary colonizers in indoor environments with their a_w_ requirement of <0.8 [12]. *A. sydowii*, a common indoor mold [13], is halotolerant, needs a high nutrient level for growth e.g., isolated from dust, gypsum wall board, ceiling, textile and wooden items in indoor environments [13,18], and is mesophilic (growth range 22–36 °C and optimal at 30 °C) [19]. *A. penicillioides*, also a common indoor mold [13], can produce septate germlings at a_w_ of 0.585 [20], and is typically recovered from dry habitats in built environments such where house dust accumulates or audio tapes and binocular lenses are stored [21].

There are limited studies about the ecological and physiological features of *Tritirachium*. It has been isolated from the sea, plant and soil [22]. It is not a common indoor mold. 

*Rhizopus* is a genus that generally needs a a_w_ of above 0.9 for growth as tertiary colonizers and is commonly isolated indoors [6]. *Rhizopus* spp. normally demand a high nutrient level for growth on water damaged building materials such as leather, wood, and paper [14,23].

*T. harzianum*, as a tertiary colonizer [6], is cellulolytic and found commonly on cellulose surfaces e.g., wooden board [24]. Isolation from aluminum foil in water-damaged building was also reported [24], which implies that *T. harzianum* may be able to grow with relatively low nutrient levels. Its growth temperature ranges from 5–36 °C with an optimal level at 30 °C [25]. Apart from the surface texture of building materials, it was pointed out that a poor state of maintenance, aging, loading (wear and tear on the material), limited cleaning access and dirt are indicators of the capability of a material to support mold growth [26]. Gravesen et al. [26] observed that all the collected building materials with mold contamination had more than three characteristics as mentioned above. 

### 3.4. Mold Contamination Scenarios

Based on the mold speciation and environmental conditions, the mold contamination scenarios were deduced. The presence of *T. harzianum* justified that the wall (M001) has been water damaged for a long period of time to support the growth of this tertiary colonizer. Water condensation was likely on this wall because the adjacent room kept air-conditioning while the air-conditioning was off after working hours in the laboratory. This cooler wall surface as compared to the room air was prone to condensation. The laboratory door is near to this wall. The infiltrated hot and humid air could easily condense on this surface. During our investigation, the air temperature and RH in the corridor were 24 °C and 96.0%, respectively, which indicate that water vapor could easily condense on the wall surface. The high abundance of *Cladosporium* spp. and the measured a_w_ of 0.75 on the surface suggest that the surface continued to dry when the air-conditioning system was in operation but the water availability was still sufficient to support the mold growth. *Cladosporium* spp., but not *T. harzianum*, were found on the bench surface (M002). This may be associated with the lower a_w_ of the bench surface than the wall (a_w_ 0.8–0.9) and different nutrients present in these sites. The bench was dusty which may support the mold growth even at a low a_w_ because the dust may create a hygroscopic environment to absorb moisture and provide nutrients. 

The water bath (M003) was slightly contaminated by *C. cladosporioides* when compared to other areas. Organic matter on the water bath surface is one of the important requirements for mold growth on metal surface. In addition, the water bath was close to the door and its position increased the chances of condensation on the water bath surface.

Interestingly, the shaker (M004) was the only area that without *Cladosporium* spp. contamination but with *A. penicillioides* and high population of *A. sydowii* (50% in this area, other areas contained 0.45–15%). These results imply that this contamination area had a low a_w_ that can only support the primary colonizer. Metal surface is unfavorable for mold growth because of the non-porous texture and lack of nutrients. Therefore, the presence of organic matter is critical to support the mold growth. The shaker did not look dusty but the flasks placed in the shaker could carry dirt that provided nutrients to certain areas.

As for the dusty sites, the packing film (M005), mineral oil box (006) and toolbox (M007), more than three types of mold species were identified on the packing film and toolbox and, although the same was true for the mineral oil box, the mold density was low. These results indicate some table-top molds rather than colonizing molds were present on the surfaces. 

*Tritirachium* sp. is not a common indoor mold and its presence on the balance (M008) relates to the unique organic dust, e.g., powder of various culture media deposited on the back of the balance. This powder is usually hygroscopic and nutritious to support mold growth. 

## 4. Discussion

### 4.1. Health Effects of the Isolated Molds

Some of the isolates could cause pathogenic or allergenic effects. *Cladosporium* was reported to be the highest occurring genus in residences of allergy patients and known to produce allergens [6,14]. Studies point out that *C. cladosporioides* has the potential to occasionally infect the human lungs, skin, eyes and brain [27]. Besides *Cladosporium* spp., *A. sydowii* was the third most prevalent species, which was reported to produce fungal volatile organic compounds (FVOCs) that may cause headache, inattentiveness and dizziness to occupants [28]. *A. sydowii* is also regarded as a pathogenic fungus that can cause aspergillosis, onychomycosis and keratomycosis in human [29]. Moreover, some *Tritirachium* spp. have been occasionally isolated from nail infections [30]. *R. microsporus* is known as a pathogenic fungus to humans and once caused the outbreak of intestinal infection in a Hong Kong hospital [31]. Although no laboratory user complained about the mold contamination and health effect, it is important to remediate and prevent recurrence of the mold contamination. 

### 4.2. Mold Control Measures 

#### 4.2.1. Maintain RH to 70%

According to the WHO recommendations, one of the main measures to control mold growth indoors is by maintaining the RH to less than 70% to limit the water supply [6]. When the air-conditioning system was in operation, the laboratory RH and temperature were at 60.5% and 22.0 °C compared to the RH of the corridor at 96% and temperature at 24.0 °C. In tropical and subtropical areas like Hong Kong, atmospheric RH can be extremely high during the humid season in March and April. For example, there were altogether 28 days during which the daily mean RH was higher than 90% in March 2016. In this study, turning on the air-conditioning system for 24 h could maintain a low RH environment and prevent mold growth. 

RH can also be reduced by using dehumidifiers with a lower energy cost than air-conditioners. However, it may still not be environmentally-friendly to operate it continuously to prevent mold contamination. Laarhoven et al.’s study [32] showed that the hyphal growth of *Penicillium rubens* can be interrupted by using a period of low RH [32]. Further research may help determine the minimum period of low RH and its maximum interval for effective interruption of mold growth. 

#### 4.2.2. Increase Room Temperature of the Adjacent Room 

Even if the RH is well controlled at 70% using dehumidifiers, mold contamination can still happen in this laboratory due to water condensation on the wall with lower temperature than the room air. The air temperature in the corridor was 28 °C, a common temperature in the summer time in Hong Kong. After the air conditioning system is turned off, the temperature in the laboratory will go up to the same level as the corridor air. The dew point for air at 28 °C and RH at 70% is 22 °C. Based on our measurement on the wall surface (M001) (21.8 °C) and the room temperature set behind the wall (22 °C), this implies that water condensation can easily happen. A sustainable solution to resolve this problem is to set the adjacent room temperature to a higher level; the Hong Kong Indoor Air Quality Scheme suggests setting the room temperature to 25 °C in the summer time to save energy [33]. This solution will save energy in the adjacent room and also reduce the chance of water condensation on the wall. As seen in our data, the wall on the right side, where the office behind the wall had the same temperature profile as the laboratory, did not have any mold contamination. Condensation provides readily available water to molds, which can significantly speed up mold growth at a much faster rate than molds obtaining water from humid air. According to the Updated VTT model, with a 1% increase in RH, the time needed to reach a visible mold level is shortened by 16 h at 28 °C [5]. The growth rate of mold will further accelerate when water condensation happens [4].

#### 4.2.3. Reduce Warm and Humid Air Infiltration 

Most of the mold contamination sites were found near the laboratory door, bench surface (M002), water bath (M003), shaker (M004) and toolbox (M007), where the warm and humid air enters the laboratory. Even if the door is closed, air can still infiltrate the laboratory through the door gap. This study clearly showed that this warm and humid air could easily condense on various surfaces. To reduce infiltration, one cost effective solution could be to seal the gaps in windows and doors. In addition, when designing the ventilation system, the airflow of the laboratory in relation to the outside air should also be considered. Items in hidden locations, packing film (M005), the mineral oil box (M006) and toolbox (M007), were also prone to condensation after the air-conditioning system was turned off, and the condensed water was more difficult to shift when the air-conditioning system was turned on again. Moreover, the lack of light exposure in these hidden locations could also favor mold growth [34].

#### 4.2.4. Control Nutrient for Mold Growth

As with other heterotopic organisms, mold requires organic carbon and nitrogen sources for growth. Ojanen et al. [5] states that mold resistant materials such as glass and metal items will not be contaminated by mold unless they are soiled. When the substrate level increases, the water requirement for mold growth decreases [5]. Sedlbauer [4] demonstrated that a mold, which requires at least 90% of RH to germinate in a day on biologically recyclable building materials, was able to germinate at 85% of RH with the supply of nutrient. A study by Laarhoven et al. [32] also showed that mold spores could start germination on gypsum substrates with Czapek Dox Broth within 20 h of incubation, which is significantly faster than on gypsum substrates alone. Therefore, once nutrient is present on surfaces, the surface becomes mold sensitive material regardless of its original nature such as on the surface of metal and plastic items in this study. Restricting the availability of nutrient is also critical to prevent mold growth especially when moisture control is hard or expensive to achieve as discussed above. The analytical balance (M008) was soiled by the powder of culture media used to grow bacteria and fungi. This powder is nutritious and hygroscopic. Both factors favor mold contamination. This analytical balance is the only mold-contaminated area on the right-hand side of the laboratory, which implies that removing this organic dust is the most effective way to prevent mold contamination in this scenario. Furthermore, nutrient control through avoiding the use of mold sensitive materials such as wood and paper could also help prevent mold contamination.

## 5. Conclusions

Indoor environments in tropical and sub-tropical regions are prone to mold contamination. Different kinds of molds can inhabit in the same room and flourish due to different microscopic environmental conditions. Building sustainability plans should regard mold hygiene as a key consideration in these regions. Although operating air-conditioners for 24 h is the most convenient way to prevent mold contamination from the user’s perspective, there are other measures that should be considered in order to prevent mold growth sustainably.

## Figures and Tables

**Table 1:** Primers used for mold identification in this study [9,10].

Species	Assay Name	Forward Primer (5′-3′)	Reverse Primer (5′-3′)
*Aspergillus penicillioides*	Apeni2	ApeniF2: CGCCGGAGACCTCAACC	ApeniR2: TCCGTTGTTGAAAGTTTTAACGA
*Aspergillus sydowii*	Asydo3	AsydoF1-1: CAACCTCCCACCCGAGAA	AversR1-1: CCATTGTTGAAAGTTTTGACTGATCTTA
*Cladosporium cladosporioides*, *svar. 2*	Cclad2	Cclad2F1: TACAAGTGACCCCGGCTACG	CcladR1: CCCCGGAGGCAACAGAG
*Trichoderma harzianum*	Tharz	TharzF1: TTGCCTCGGCGGGAT	TharzR1: ATTTTCGAAACGCCTACGAGA
*Rhizopus azygosporus/microsporus*		Rh1: TTTCCAGGCAAGCCGGACCG	Rh2: TATTCCCAGCCAACTCGCCAAAT

**Table 2:** Environmental information and conditions in the sample sites.

Label	Site/Object	Surface	Surface Area (cm^2^)	a_w_	Temp. (°C)	Location	Light Level	Previously Cleaned	Mold Patch Area (cm^2^)	Visible Dust
M001	Wall	Anti-mold painted cement brick wall	60,000	0.75	21.8	Open	Dim	Bleach	120	No
M002	Bench	Thermosetting high-pressure laminates	42,000	0.74	21.7	Hidden	Dim	No	400	Yes
M003	Water bath	Metal	6750	0.71	20.1	Open	Room	Bleach	69	Yes
M004	Shaker	Metal	6500	0.71	19.8	Open	Room	No	150	No
M005	Packing film	Plastic	5200	0.69	21.3	Hidden	Dark	No	1100	Yes
M006	Mineral oil box	Plastic	810	0.69	21.4	Hidden	Dark	No	225	Yes
M007	Toolbox	Plastic	5800	0.73	21.1	Hidden	Room + sunlight	No	270	Yes
M008	Analytical balance	Metal	2714	0.65	21.2	Open	Dim	No	70	No

**Table 3:** Ecological niches and growth requirements of the isolated molds.

Isolated Molds	Ecological Niches and Growth Requirements	Reference
*Cladosporium* spp*.*	Secondary colonizers, generally require 0.8–0.9 a_w_ for growth	[12]
*Cladosporium cladosporioides*	Common indoor mold, requires > 0.87 a_w_, needs a high level of organic matter, psychrophilic	[13,14,15]
*Cladosporium halotolerans*	Requires > 0.82 a_w_, can grow under a low level of organic matter, psychrotolerant	[16,17]
*Aspergillus* spp.	Primary colonizers, <0.8 a_w_	[12]
*Aspergillus sydowii*	Common indoor mold, requires > 0.793 a_w_, needs a high level of organic matter, halophilic, mesophilic	[13,18,19]
*Aspergillus penicillioides*	Common indoor mold, xerophilic (can grow even at 0.585 a_w_), could be associated with water damaged buildings	[13,20,21]
*Tritirachium* sp.	Not a common indoor mold, needs a high level of organic matter, halotolerant	[22]
*Rhizopus* spp*.*	Tertiary colonizers, require > 0.9 a_w_, normally need a high level of organic matter for growth	[6,14,23]
*Trichoderma harzianum*	Common indoor mold, tertiary colonizer, requires > 0.9 a_w_, cellulolytic, requires a high level of organic matter, mesophilic, psychrotolerant	[24,25]
